# Effectiveness of psychoeducation interventions for pregnant women with gestational diabetes mellitus: an integrative review

**DOI:** 10.1186/s12889-024-20428-6

**Published:** 2024-10-22

**Authors:** Mutairah Alshammari, Regina Lai Tong Lee, Michelle Stubbs, Sally Wai-Chi Chan

**Affiliations:** 1 School of Nursing and Midwifery, College of Health, Medicine and Wellbeing, The University of Newcastle, University Drive, Callaghan, NSW 2308 Australia; 2 Nursing Department, College of Applied Medical Sciences, Jouf University, King Khalid Road, Sakaka, Kingdom of Saudi Arabia; 3 The Nethersole School of Nursing, Faculty of Medicine, The Chinese University of Hong Kong, Shatin, Hong Kong SAR China; 4 Tung Wah College, Homantin, Hong Kong SAR China

**Keywords:** Gestational diabetes Mellitus, Self-management, Self-efficacy, Health education, Psychoeducation, Pregnancy

## Abstract

**Background:**

Gestational diabetes mellitus occurs in approximately 15–17% of pregnant women worldwide and causes high mortality and morbidity for mothers and infants. Pregnant women who are newly diagnosed with gestational diabetes mellitus experience higher levels of stress and anxiety than pregnant women without this condition. Thus, it is important to identify effective interventions to help pregnant women cope with the additional stress and anxiety associated with pregnancy-related complications.

**Aim:**

This integrative review aimed to synthesise evidence on the effects of educational interventions for pregnant women with gestational diabetes mellitus regarding knowledge, self-efficacy, self-care behaviour, anxiety, depression, and birth outcomes.

**Methods:**

An integrative review of articles published between 2009 and 2024, written in English and Arabic. The review followed the Whittemore and Knafl’s 5-stage process framework.

**Results:**

From the 922 abstracts identified using search terms, 16 articles were eligible for this review. Psychoeducational interventions were provided for (1) informational support: information about gestational diabetes mellitus, diabetes mellitus, blood glucose monitoring, exercise management, diet management, and stress; (2) motivational support: setting individual goals, enhancing health behaviours, and motivational messages; (3) emotional support: expression of feelings, enforcement of self-management, and sharing of experiences; and (4) relaxation techniques: breathing exercises, meditation, and mindfulness. In this review, only two studies entirely focused on reducing stress and anxiety through cognitive-behavioural stress management training and mindfulness training. The effects of the interventions on self-efficacy, knowledge, depression, anxiety, and birthing outcomes were inconsistent due to variations in intervention designs and duration. However, consistent positive outcomes were found in self-care behaviours.

**Conclusion:**

This integrative review found informational and motivational support were frequently used by pregnant women. In contrast, emotional support and relaxation techniques were rarely used. Psychoeducational interventions may enhance self-care behaviours, improve self-efficacy, and reduce stress and depression for women with gestational diabetes mellitus. Nurses and midwives play an essential role in providing holistic care through comprehensive psychoeducational interventions for pregnant women.

## Introduction

Gestational diabetes mellitus (GDM) is the most common metabolic complication of pregnancy and contributes to high mortality and morbidity rates in mother and infants. GDM is defined as “any degree of glucose intolerance with onset or first recognition during pregnancy” [1]. In 2021, the global prevalence of GDM in pregnant women was predicted to be approximately 16.7% [2], with considerable differences between nations owing to variations in diagnostic criteria, screening methods, and population characteristics. GDM causes serious short and long-term health complications for both mothers and infants. For example, women may have gestational hypertension, preeclampsia, require a caesarean section, and increased risk of developing type 2 diabetes [3]. Their infants have increased risk of hypoglycaemia, macrosomia, prematurity, birth trauma, and developing type 2 diabetes and obesity later in life [3].

Current GDM management strategies aim to control glycaemic levels through an appropriate glycaemic index diet, lifestyle modifications, and pharmacological treatments [4]. Pregnant women with GDM need to perform self-care consisting of daily self-monitoring of blood glucose, meet daily nutritional needs, engage in regular exercise, and adjust insulin doses when necessary [5]. However, several factors influence pregnant women with GDM self-care behaviours such as knowledge, self-efficacy, emotions and physical skills [5].

Women with GDM may experience stress and anxiety because of this diagnosis [6, 7]. They may also have difficulties following the required restrictive diet and appropriate exercise plans during pregnancy [6, 7]. Furthermore, they may require additional antenatal visits and tests, which may increase the burden of pregnancy. Pregnant women with GDM experiencing uncontrolled glucose levels, high levels of stress and anxiety have a higher risk of adverse outcomes; relating to mode of birth, and maternal and neonatal health than pregnant women without GDM [7]. Therefore, psychoeducational programs should include strategies to reduce stress, anxiety and support pregnant women with GDM to potentially improve their physical and mental outcomes.

Psychoeducational interventions are psychosocial strategies for behavioural changes, including problem-solving skills, social skills training, and self-care skills [8]. This educational approach has been designed to improve patients’ knowledge and understanding of their conditions, treatment, and coping skills [8]. In 2015, a systematic review focused on managing diabetes-related outcomes and the efficacy of psychological interventions, such as motivational interviewing, cognitive behavioural therapy, brief psychodynamic psychotherapy, and client-centered therapy, reported that psychological interventions significantly reduced haemoglobin A1C, depression, and anxiety in patients with type 2 diabetes [9]. Psychoeducational interventions using motivational and cognitive counselling, such as problem-solving training, expression of feelings, and personal feedback, played a significant role in controlling glycaemic levels, managing depressive symptoms, and enhancing overall well-being in patients with type 2 diabetes [10]. Thus, psychoeducational interventions may be helpful for pregnant women with GDM.

Current interventional studies have focused on the effectiveness of lifestyle changes, such as adopting healthy diet, and practicing appropriate regular exercises, taking oral or injectable medications, and taking supplements [11]. A Cochrane review concluded that there was inconclusive evidence about the impact of these interventions for women with GDM [11]. This review also highlighted the lack of high-quality studies relating to GDM interventions and emphasised the need for further rigorous trials. However, there is currently a lack of interventional studies that primarily aim to reduce stress and anxiety levels for women with GDM.

### Objective

This integrative review aimed to synthesise evidence on the effects of educational interventions, including information, guidance, and support for GDM and/or for psychological health of pregnant women with GDM.

## Methods

### Protocol

This integrative review followed the framework introduced by Whittemore and Knafl [12]. The framework includes a five-stage process; (1) problem identification, (2) literature search, (3) data extraction and evaluation, (4) data analysis, and (5) presentation of the findings. The integrative review protocol was prepared using the method outlined in the Preferred Reporting Items for Systematic Reviews (PRISMA) [13]. Psychoeducational intervention was defined as interventions that provided information, guidance, and support for GDM and/or for psychological health.

### Eligibility criteria

- Articles in English or Arabic only (other languages were not included due to lack of funding for translation).
- Primary published research. Systematic review articles were also searched for further publications; however, they were not included in this integrative review.
- Studies with pregnant women diagnosed with GDM.
- Studies that examined single- or multi-component educational interventions and involved comparison groups (randomised controlled trials, experimental or quasi-experimental studies, and pre and post studies).
- Studies that measured one or more of the following outcomes: knowledge, self-efficacy, self-care behaviour, anxiety, depression, preterm birth, birth type, admission to the neonatal intensive care unit (NICU), and infant birth weight.

### Exclusion criteria

- Studies with participants diagnosed with type 1 or 2 diabetes unrelated to pregnancy.
- Studies that included only pharmacological interventions.
- Studies that were not primary research, such as case reports, conference abstracts, textbooks, commentaries, care guidelines, theses, dissertations, and grey literature.
- Studies that did not meet the Academy of Nutrition & Dietetics methodological quality score.

## Stage 1: problem identification

The problems addressed by this review were:

1. What educational interventions were conducted for pregnant women with GDM?
2. What was the effectiveness of these interventions on knowledge, self-efficacy, self-care behaviour, anxiety, depression, and birth outcomes?

## Stage 2: literature search

### Search Strategy

The following keywords were used for the search: (gestational diabetes OR GDM OR Diabetes in pregnancy) AND (intervention OR psychoeducation OR psycho-education OR psycho education OR education OR exercise OR physical activity OR diet OR strategy* OR program* OR management OR treatment* OR therapy* OR counsel* OR training OR instructions OR advice OR self-management) AND (self-efficacy OR knowledge OR self-care behaviour OR anxiety OR depression OR preterm birth OR birth weight OR caesarean section OR NICU OR birth type) AND (randomised controlled trial OR random* OR experimental study* OR pre and post study*).

#### Information sources

**Fig. 1:**
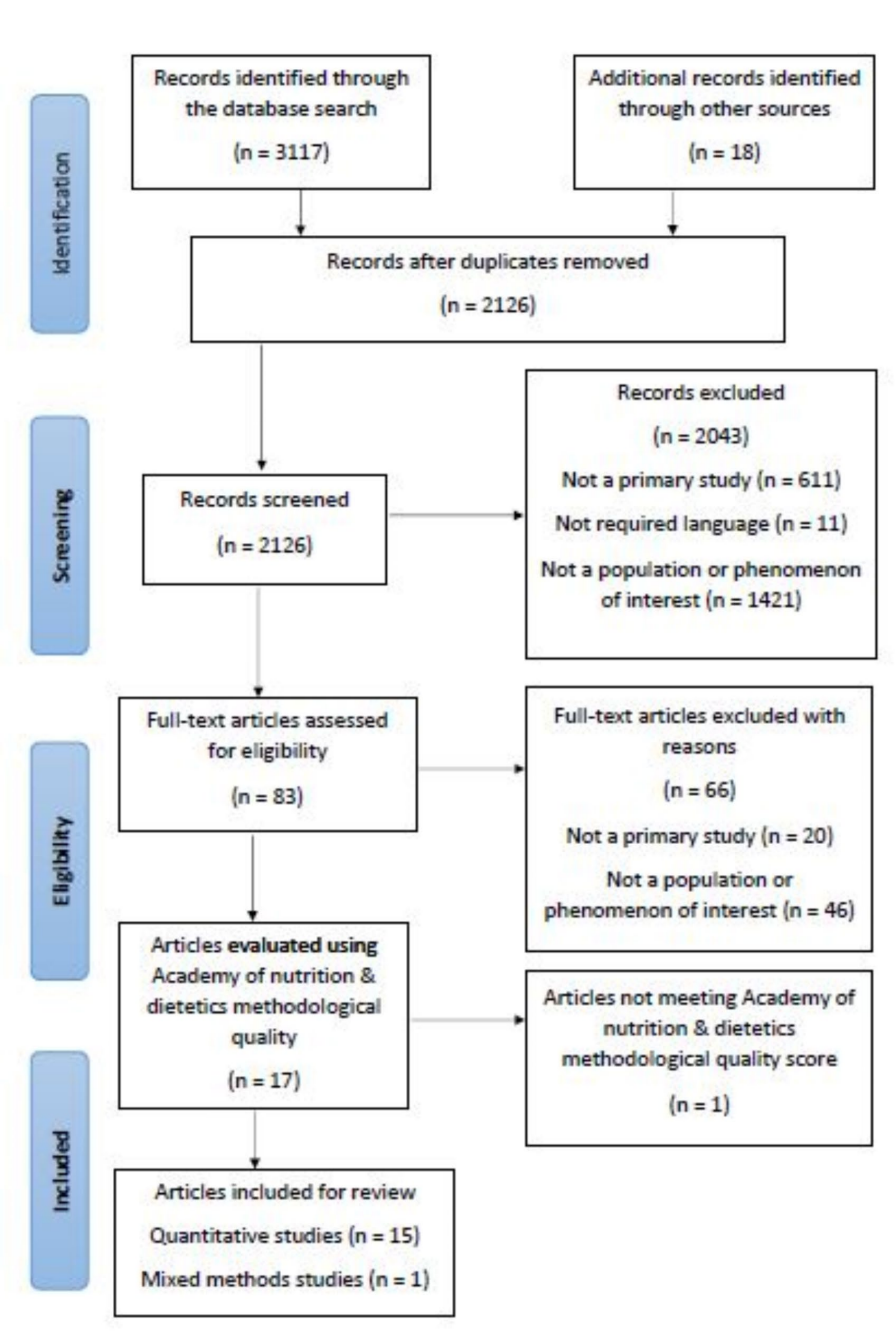
PRISMA flow diagram

A computerised literature search was conducted using the following electronic databases: MEDLINE, CINAHL, Cochrane Library, PsycINFO, Maternity and Infant Care Database (MIDIRS), and Saudi Digital Library (SDL). SDL was searched as the first author is a native speaker of Arabic. A manual search was performed for relevant articles among the references of the retrieved articles. The literature search was conducted from November 2019 to June 2024 to locate updated evidence and account for the new International Association of Diabetes and Pregnancy Study Group (2010) screening and diagnosis criteria for screening and diagnosis GDM [14]. Figure 1 shows a PRISMA flow diagram.

#### Selection of sources of evidence

Data were extracted using Endnote, and duplicate articles were removed. The authors (MA and MS) independently evaluated the titles and abstracts. Articles that did not meet the eligibility criteria were also excluded. The authors (MA and MS) also evaluated the remaining full articles. The first author (MA) then summarised them in a table for the other authors to discuss. Subsequently, all authors discussed results to reach a consensus, with final sources for inclusion agreed upon and synthesised in Table 1.

**Table 1:** Quality appraisal for included studies

	Al-Hashmi et al.,	Cao et al.,	Carolan-Olah & Sayakhot	Draffin et al.,	Homko et al.,	Kim et al.,	Kolivand et al.,	Mirghafourvand et al.,	Mohebbi, et al.,	Sayakhot, et al.,	Sen & Sirin	Sen & Sirin	Viswanath	Yang et al.,	Zaheri et al.,	Zandinava, et al.,	Zeinabeh et al.,
Overall quality rating	*P*	N	*P*	*P*	R	R	*P*	*P*	*P*	*P*	R	R	R	*P*	*P*	*P*	*P*
Q1	Y	Y	Y	Y	Y	Y	Y	Y	Y	Y	Y	Y	Y	Y	Y	Y	Y
Q2	Y	N	Y	Y	Y	Y	Y	Y	Y	Y	Y	Y	Y	Y	Y	Y	Y
Q3	Y	N	Y	Y	N	N	Y	Y	Y	Y	UC	UC	N	Y	Y	Y	Y
Q4	NA	N	N	UC	Y	N	Y	NA	NA	N	UC	UC	UC	Y	Y	NA	Y
Q5	N	N	N	N	N	N	Y	N	N	N	N	N	N	Y	Y	N	UN
Q6	Y	UC	Y	Y	Y	Y	Y	Y	Y	Y	Y	Y	Y	Y	Y	Y	Y
Q7	Y	Y	Y	Y	Y	Y	Y	Y	Y	Y	Y	Y	Y	Y	Y	Y	Y
Q8	Y	Y	Y	Y	Y	Y	Y	Y	Y	Y	Y	Y	Y	Y	Y	Y	Y
Q9	Y	N	Y	Y	N	Y	N	Y	Y	Y	Y	Y	N	N	Y	Y	Y
Q10	Y	Y	Y	Y	Y	Y	Y	Y	Y	Y	Y	Y	Y	Y	Y	Y	UC

Q1: Was the research question clearly stated?; Q2: Was the selection of study subjects/patients free from bias?; Q3: Were study groups comparable?; Q4: Was method of handling withdrawals described?; Q5: Was blinding used to prevent introduction of bias?; Q6: Were intervention/therapeutic regimens/exposure factor or procedure and any comparison(s) described in detail? Were intervening factors described?; Q7: Were outcomes clearly defined and the measurements valid and reliable?; Q8: Was the statistical analysis appropriate for the study design and type of outcome indicators?; Q9: Are conclusions supported by results with biases and limitations taken into consideration?; Q10: Is bias due to study’s funding or sponsorship unlikely?; Y: Yes; N: No; NA: Not Applicable; UC: Unclear; P: Positive rating; N: Negative rating; R: Neutral rating

## Results

This review initially aimed to collect and synthesise articles examining the effects of psychoeducational interventions on women with GDM. However, only one psychoeducation intervention was found; therefore, the search terms were broadened to include more educational interventions as they might have integrated psychoeducational elements. A subsequent search of electronic databases revealed 1,069 articles. An additional 18 articles were identified from the manual search of reference lists and systemic review articles. After eliminating duplicate articles, 922 studies were identified. After reviewing titles and abstracts, 860 papers were removed, and 62 full text articles were screened against the inclusion criteria. Seventeen articles were eligible and included in the final review before quality assurance based on the inclusion and exclusion criteria. Of these, four articles reported findings from two different interventions and were included because they reported different outcomes. Arabic databases were searched, however, none of the included studies were in Arabic.

## Stage 3: data evaluation

### Quality appraisal

The methodological quality of the included studies was assessed using the Academy of Nutrition and Dietetics Quality Criteria Checklist for Primary Research [15]. This checklist includes four relevance and ten validity questions. Each article must fulfil the four validity criteria to obtain a positive score in quality rating. These four questions were related to selection bias, group comparability, intervention or exposure factor description, outcomes, and measurement definitions. Answers were recorded as follows: “Yes”, meeting the criteria; “No”, failing to meet the criteria; or “Unclear”, if the criteria were not clearly reported. The articles were rated as: positive (+) if the specific four validity questions and one more question were answered as “Yes”, negative (-) if six or more of the validity questions were answered as “No”, and “Unclear” if the four specific validity questions did not indicate that reports were exceptionally strong.

Eleven studies provided sufficient information regarding selection bias, group comparability, intervention or exposure factors, outcomes, and measurement definitions; and received a positive quality rating [16–26]. On the other hand, five studies were rated as neutral, as the answers to the four validity questions were not appropriately reported, indicating that these studies were not exceptionally strong [27–31]. The main issue identified in the reviewed studies was the lack of information regarding the randomisation process.

One study received a negative rating owing to a lack of details on the intervention content, duration, and randomisation process [32], and was excluded. Thus, sixteen studies were finally included in the review (Fig. 1); their quality assessments are presented in Table 1.

## Stage 4: data analysis

### Data extraction, analysis and synthesis

Following systematic review reporting guidelines, a data extraction form was used to facilitate the data extraction process. Extracted data included information on the study (author, publication year, paper title, and paper purpose), the study methods (study design, sampling, and sample size, methods, and outcomes measures). The first author (MA) did the initial extraction, and the others checked the accuracy. Deductive content analysis was used to analyse the data. We read the full-texts of the selected papers to obtain an understanding of the content. The data was then tabulated and synthesised. A summary of the selected studies is presented in Table 2.

**Table 2:** Sample and study characteristics

First author /year	Country	Study Design	Study Group (s)	Sampling
**Al-Hashmi et al.**,** 2018**	Oman	Randomised quasi-experimental study	IG (*n* = 45) CG (*n* = 45)	Pregnant women with GDM Mean age: 33.5 ± 5.1 years Age range: 19–43 years old Mean BMI: 29.0 ± 7.0 Personal history of GDM: 38.9% Family history of GDM: 32.2% Mean parity: 2.3 ± 1.8 History of baby birth weight of > 4 kg: 2.2%
**Carolan-Olah and Sayakhot**,** 2019**	Australia	RCT	IG (*n* = 52) CG (*n* = 58)	Mean age: 31.7 years Age range: 19–43 years old BMI rang: > 19.5 kg/m ^2^ - > 50.0 kg/m ^2^
**Draffin et al.**,** 2017**	UK	RCT	IG (*n* = 75) CG (*n* = 67)	Age range: 19–44 years Gestational age at diagnosis: IG: 27.5 (4.6), CG: 26.8 (4.5)
**Homko et al.**,** 2012**	US	Randomised quasi-experimental study	IG (*n* = 36) CG (*n* = 38)	Age: IG: 30.3 ± 6.0, CG: 30.0 ± 7.5 BMI: IG:34.1 ± 8.5, CG: 34.1 ± 9.8 Parity: IG: 1.7 ± 1.8, CG: 1.1 ± 1.0
**Kim et al.**,** 2019**	South Korea	Quasi-experimental study	IG (*n* = 22) CG (*n* = 22)	Age: IG: 35.1 ± 3.8, CG: 36.4 ± 3.1 Prim-parity: IG: 77.3%, CG: 90.9% Gestational age (weeks): IG: 27.4 ± 1.6, CG: 27.4 ± 1.3 BMI (kg/m^2^): IG: 25.0 ± 2.7, 25.4 ± 3.6
**Kolivand et al.**,** 2019**	Iran	RCT	IG (*n* = 75) CG (*n* = 76)	Age: IG: 32.6 ± 5.7, CG: 30.2 ± 4.5 Parity: IG: 1.0 ± 0.9, CG: 0.7 ± 0.8 BMI (kg/m^2^): IG: 27.1 ± 4.3, CG: 26.1 ± 3.3 Gestational age (weeks): IG: 26.7 ± 5.2, CG: 27.3 ± 2.7 History of GDM: IG: 13.4%, CG: 23.7%
**Mirghafourvand et al.**,** 2019**	Iran	RCT	IG(*n* = 46) CG (*n* = 46)	Age: IG: 30.3 ± 5.1, CG: 31.7 ± 4.8 BMI, kg/m^2^: IG: 26.7 ± 3.0, CG: 27.6 ± 3.5 Prim-parity: IG: 30.4%, CG: 41.3%
**Mohebbi et al.**,** 2019**	Iran	Randomised quasi-experimental study	IG *n* = 55) CG (*n* = 55)	Mean age: IG: 30.7 ± 6.53, CG: 30.78 ± 6.79 BMI: IG: 27.84 ± 2.87, CG: 27.63 ± 4.42
**Sayakhot et al.**,** 2016**	Australia	RCT	IG (*n* = 56) CG (*n* = 60)	Age range: IG: 31–35 years CG: 26–30 years
**Sen and Sirin**,** 2014**	Turkey	Non-randomised quasi experimental study	IG (*n* = 30) CG (*n* = 30)	Age range: 25- > 35 years
**Sen and Sirin**,** 2015**	Turkey	Non-randomised quasi experimental study	IG (*n* = 30) CG (*n* = 30)	Mean age: IG: 32.33 ± 4.22, CG: 32.96 ± 4.38
**Viswanath et al.**,** 2014**	India	Randomised quasi-experimental study	IG (*n* = 20) CG (*n* = 20)	Mean age: IG: 30.5 ± 5.3, CG: 29.9 ± 4.6
**Yang et al.**,** 2014**	China	RCT	IG (*n* = 339) CG (*n* = 361)	Age: IG: 29.9(3.5), CG: 29.7(3.2) BMI: IG: 22.9(3.6), CG:23.4(3.9) Nulliparous: 95.1% Gestational age: IG: 10.8(2.4), CG: 10.8(2.3)
**Zaheri et al.**,** 2016**	Iran	RCT	IG (*n* = 40) CG (*n* = 40)	Mean age: IG: 26.95 (3.65), CG: 26.25 (5.16) Gestational age: IG: 27.77 (2.34), CG: 27.95 (2.12)
**Zandinava et al.**,** 2017**	Iran	RCT	IG (*n* = 46) CG (*n* = 46)	Mean Age: IG: 30.3 (5.1), CG: 31.7 (4.8) History of GDM: 2%
**Zeinabeh et al.**,** 2023**	Iran	Randomised quasi-experimental study	IG (*n* = 40) CG (*n* = 40)	Mean age: IG: 28.84 ± 6.20, CG: 29.03 ± 5.42 BMI: IG: 26.52 ± 5.38, CG: 26.12 ± 5.38

BMI: Body mass Index; CG: Control Group; GDM: Gestational Diabetes Mellitus; IG: Intervention Group; RCT: Randomized Controlled trial

## Stage 5: presentation

### Characteristics of studies

As shown in Table 1, the study designs of the sixteen studies were: randomised controlled trials (RCT) (*n* = 8), randomised quasi-experimental studies (*n* = 5), non-randomised quasi-experimental studies (*n* = 2), and mixed methods experimental pre- and post-studies (*n* = 1). The included studies compared the control groups of only routine care and intervention groups. The sample size in the studies ranged from 22 to 700 women diagnosed with GDM. The studies were conducted in: Iran (*n* = 6), Australia (*n* = 2), Turkey (*n* = 2), the United Kingdom (*n* = 1), the United States of America (*n* = 1), China (*n* = 1), India (*n* = 1), Oman (*n* = 1), and South Korea (*n* = 1). Table 2 presents the sample and study characteristics.

### Interventions

#### Content of the interventions

Most of the included interventional studies focused on promoting lifestyle changes, such as blood glucose monitoring, appropriate exercises, and diet [16–23, 25, 27–31]. Only three studies provided stress reduction interventions using cognitive-behavioural stress management training and mindfulness training [19, 24, 26]. Table 3 presents the intervention contents.

**Table 3:** Intervention contents

Study author	Intervention type	Intervention details	Duration	Delivery mode
**Al-Hashmi et al.**,** 2018**	• Informational support • Self-management motivation	• Give information about gestational diabetes and gestational diabetes related maternal and neonatal complications • Give information about the importance of healthy lifestyle behaviours (healthy diet, safe exercise and maintaining self-monitored blood glucose levels to prevent gestational diabetes complications) • Prevention of type 2 diabetes • Writing specific and measurable goals to adhere to healthy behaviours • Role modelling • Receiving short biweekly motivational text messages	4 weeks	• Group face-to-face sessions • An educational video • A pamphlet • Booster phone call
**Carolan-Olah and Sayakhot**,** 2019**	• Informational support	• Give information about healthy food choices • Give information about healthy habits/healthy lifestyle • Give information about testing blood glucose levels • Give information about emotions and family	6 months	• Web-based educational intervention
**Draffin et al.**,** 2017**	• Informational support • Self-management motivation • Emotional support	• Give information about gestational diabetes • Diet management • Blood glucose management • Weight management • Insulin therapy • Prevention of type 2 diabetes • Enhancing positive health behaviours • Promoting positive feelings toward outcomes for woman and baby	6 months	• Educational DVD
**Homko et al.**,** 2012**	• Informational support • Self-management motivation • Emotional support	• Receiving educational message/tip • Explain how to use educational application • Reminders to record and transmit clinical data in the system (blood glucose levels, foetal movement counting, insulin doses and episodes of hypoglycaemia) • Receiving reinforcement regarding gestational diabetes self-management • Receiving feedback and emotional support	3 months	• Telemedicine system
**Kim et al.**,** 2019**	• Informational support • Self-management motivation	• Give information about diet management • Explain how to use educational application • Setting a goal for management • Checking diet management • Checking exercise management • Checking glucose monitoring • Checking weight management • Enhancing positive health behaviours	12 weeks	• Online self-care program
**Kolivand et al.**,** 2019**	• Informational support • Self-management motivation • Relaxation techniques	• Give information about diabetes • Give information about nutrition • Give information about self-monitoring of blood glucose • Give information about physical activity and exercise • Give information about Insulin • Give information about mental health and pregnancy • Give information about delivery and postpartum care • Checking diet management • Checking exercise management • Checking glucose monitoring • Checking weight management • Relaxation exercise exercises	4–7 weeks	• Face-to-face educational sessions • Self-care guidebook • Educational software • Phone call
**Mirghafourvand et al.**,** 2019**	• Informational support	• Give information about diabetes and its types • Give information about gestational diabetes, its complications for the mother and the foetus • Give information about the concept of self-care • Give information about diet • Give information about exercise • Give information about measurement of blood glucose • levels at home, and normal levels of blood glucose • Give information about prevention of infection, and foot care	3 months	• Group face-to-face sessions • A booklet
**Mohebbi et al.**,** 2019**	• Informational support • Self-management motivation	• Give information about gestational diabetes • Give information about diet and healthy lifestyle • Setting a goal for management • Motivational interviewing • Checking glucose monitoring • Enhancing positive health behaviours	4 weeks	• Group face-to-face sessions • Booster phone call
**Sayakhot et al.**,** 2016**	• Informational support	• Give information about healthy food choices • Give information about healthy habits/healthy lifestyle • Give information about testing blood glucose levels • Give information about emotions and family	15–30 min	• Web-based educational intervention
**Sen and Sirin**,** 2014**	• Informational support • Self-management motivation • Emotional support	• Give information about type 2 diabetes mellitus and its types • Give information about gestational diabetes, its prevalence, its pathophysiology, risks factors, maternal risks, foetal-neonatal risks • Give information about nutrition and diet, exercise, and self-blood glucose monitoring • Give information about insulin treatment • Give information about hypoglycemia • Give information about intrapartum care and postpartum care • Give information about healthy lifestyles behaviours • Give information about stress management • Demonstration and practicing skills (e.g. using insulin pen, measurement of blood glucose) • Promoting positive feelings toward outcomes for woman and baby • Enhancement of positive psychological status	4 days	• Group face-to-face sessions
**Sen and Sirin**,** 2015**	• Informational support • Self-management motivation • Emotional support	• Give information about type 2 diabetes mellitus and its types • Give information about gestational diabetes, its prevalence, its pathophysiology, risks factors, maternal risks, foetal-neonatal risks • Give information about nutrition and diet, exercise, and self-blood glucose monitoring • Give information about insulin treatment • Give information about hypoglycemia • Give information about intrapartum care and postpartum care • Give information about healthy lifestyles behaviours • Give information about stress management • Demonstration and practicing skills (e.g. using insulin pen, measurement of blood glucose) • Promoting positive feelings toward outcomes for woman and baby • Enhancement of positive psychological status	4 days	• Group face-to-face sessions
**Viswanath et al.**,** 2014**	• Informational support • Self-management motivation	• Give information about self-care management • Reinforcement the self-care measures • Clarification of the doubts • Reviewing of diet log • Evaluation of self-care	4 weeks	• Group face-to-face sessions • Self-care guide
**Yang et al.**,** 2014**	• Self-management motivation	• Checking diet management • Checking exercise management • Checking glucose monitoring • Checking weight management	4 months	• Individualised counselling • Group education sessions
**Zaheri et al.**,** 2016**	• Informational support • Self-management motivation • Relaxation techniques	• Give information about stress and its management • Give information about time management, anger management • Give information about cognitive restructuring • Give information about healthy lifestyles • Problem solving skills • Coping with stress • Progressive relaxation exercises	3 weeks	• Group face-to-face sessions
**Zandinava et al.**,** 2017**	• Informational support	• Give information about gestational diabetes, its causes, symptoms, and treatment • Give information prevention of complications of gestational diabetes • Give information physical activity • Give information nutrition	4 weeks	• Group face-to-face sessions • A booklet
**Zeinabeh et al.**,** 2023**	• Informational support • Relaxation techniques	• Give information about the concept of mindfulness • Mindfulness exercises	4 weeks	• Group face-to-face sessions

From the sixteen articles, four main teaching content was identified in relation to psychoeducational elements: (1) informational support, (2) self-management motivation, (3) emotional support, and (4) relaxation techniques. The most common teaching content was self-management motivation and informational support, which were addressed in 11 studies [16, 18, 19, 21, 23, 24, 27–31]. Among them, four studies provided emotional support [18, 27, 29, 30], and three studies provided relaxation techniques [19, 24, 26]. Four studies provided only informational support [17, 20, 22, 25]. Most of the studies provided multiple intervention contents [16, 18, 19, 21, 24, 26–31]. Table 3 presents intervention contents.

Common components in selected studies that focused on self-management motivation included checking blood glucose, diet, and enhancing positive health behaviours. Other educational components include exercise management, motivational messages, writing individual goals, and providing tips and advice. Providing information about GDM, diet, exercise, blood glucose monitoring, prevention of diabetes mellitus, and weight management were the most common components in studies that provided informational support. Informational support intervention studies also included information about the mental health, emotions, family, and stress management, but did not allow time for discussion on these topics [17, 22]. Some studies, that provided emotional support, encouraged women to express their feelings in small groups, express their willingness to self-manage their GDM, express their feelings toward women and babies’ health outcomes, and provide individual phone calls to support pregnant women with GDM [18, 19, 27, 29, 30]. Finally, studies that provided relaxation techniques used breathing exercise, progressive relaxation exercises, and mindfulness and meditation sessions [19, 24, 26].

#### Mode of delivery for the interventions

The mode of delivery varied across studies. Nine studies used a single-component strategy. Two were web-based and used the same web-site link [17, 22]. One study provided a DVD for each participant [18]. Six studies provided face-to-face group education through lectures with written materials, such as booklets [20, 25, 26, 29–31].

Seven studies used multi-component interventions [16, 19, 21, 23, 24, 27, 28]. Multi-component interventions involved integrating two or more strategies to provide a single intervention. Four studies used group education and alternate strategies, such as videos, phone calls, and educational or motivational messages [16, 19, 21, 24]. One study used individualised counselling, and face-to-face group education sessions [23], and two used internet access with phone calls, feedback, messages, reminders, and online or phone educational sessions [27, 28].

#### Frequency and duration of the interventions

Not all the included studies reported the duration of the interventions; however, two hours sessions were most commonly reported, and the number of sessions varied from one to eight [16, 26, 29–31]. The total duration of interventions varied from 15 to 30 min to six months, with four weeks duration being the most frequently employed by six studies [16, 19, 21, 25, 26, 31].

#### Effects of interventions on knowledge

One non-randomised quasi-experimental study reported the statistically significant effects of face-to-face educational interventions on women’s knowledge, comparing pre- and post-intervention scores between the intervention group and the control group [29]. Two RCTs reported no significant effects of educational interventions on the knowledge of women with GDM [18, 22]. However, both studies had a methodological error as they did not measure women’s knowledge before the interventions. As a result, we were unable to determine the changes on knowledge levels and determine the effects of the interventions. Table 4 presents outcomes measures and effects.

**Table 4:** Outcomes measures and effects

Study author	Outcome	Outcome measures	Key findings
**Al-Hashmi et al.**,** 2018**	Self-efficacy Adherence to healthy behaviours	The revised 20-item Diabetes Management Self-Efficacy Scale (DMSES) The revised 11-item Summary of Diabetes Self-Care Activities (SDSCA)	There were significant positive changes in self-efficacy and adherence score between the intervention and control groups
**Carolan-Olah and Sayakhot**,** 2019**	Infant birthweight	Medical records	There were no statistically significant changes in infant weight at birth between the intervention and control groups
**Draffin et al.**,** 2017**	Anxiety Stress Self-efficacy Knowledge	The State-Trait Anxiety Inventory (STAI) Prenatal Distress questionnaire (PDQ) Diabetes Empowerment Scale (DES) A short study specific questionnaire	There were no significance difference in all study outcomes between the intervention and control groups
**Homko et al.**,** 2012**	Infant birth weight Caesarean birth Neonatal intensive care unit (NICU) admission Preterm birth	Medical records	There were no significant changes in infant birth weight, caesarean birth, NICU admission, and preterm birth between the intervention and control groups.
**Kim et al.**,** 2019**	Self-care behaviours Anxiety Depression	Adapted tool from previous studies The State-Trait Anxiety Inventory (STAI) Self-Rating Depression Scale	Self-care behaviours increased in both groups, anxiety decreased in the intervention group, and depression increased in both groups.
**Kolivand et al.**,** 2019**	Self-efficacy Birth weight Type of birth Neonatal hospitalization	Eight items self-efficacy questionnaire adapted from a previous study Medical records Medical records Medical records	There were a significant change in self-efficacy score, and neonatal hospitalization in the intervention group. However, there were no significant differences in birth weight and type of delivery
**Mirghafourvand et al.**,** 2019**	Caesarean birth Preterm labour Infant weight	The pregnancy outcomes checklist questionnaire	There were no significant changes in infant birth weight, and preterm labour between groups. However, there were statistically significant changes in caesarean birth between groups
**Mohebbi et al.**,** 2019**	Perceived self-efficacy	A questionnaire developed based on Health belief Model (HBM) constructs	There was significant changes in self-efficacy in the intervention group compared with control group
**Sayakhot et al.**,** 2016**	Knowledge of gestational diabetes Knowledge of food choices and exercise Knowledge of gestational diabetes self-management	Health questionnaires developed/adapted by previous studies	There was no significant change in knowledge in between groups
**Sen and Sirin**,** 2014**	Knowledge	Gestational Diabetes and Management Achievement Test	There was a statistically significant change in knowledge between groups
**Sen and Sirin**,** 2015**	Health behaviours Self-efficacy	The Health Promotion Lifestyle Profile II (HPLP II) Scale Self-Efficacy Scale (SES)	There was a significant increase in the health behaviours in the intervention group. There was no significant change in self-efficacy level for both groups
**Viswanath et al.**,** 2014**	Self-care agency	Gestational diabetes related self-care agency (GDSCA) scale	There was a significant change in self-care agency in the intervention group
**Yang et al.**,** 2014**	Birth weight	Medical records	Birth weight of infants in the intervention group was lower than that in the control group
**Zaheri et al.**,** 2016**	Depression Anxiety Stress	Depression, Anxiety and Stress Scale (DASS-42)	There was a significant change between two groups in stress level two weeks after intervention
**Zandinava et al.**,** 2017**	Self-care behaviours	Self-care behaviours questionnaire	There was a significant change in self-care behaviours between the intervention and the control group after adjusting for the baseline score
**Zeinabeh et al.**,** 2023**	Perceived stress	Perceived stress scale developed by a previous study	There was a significant difference in stress score between groups

#### Effects of interventions on self-efficacy

This review found inconsistent results on the effects of educational interventions on self-efficacy. Three studies (one RCT and two quasi-experimental studies) showed significant improvements in self-efficacy levels among women with GDM in the intervention group compared with the control group [16, 19, 21]. Whereas two studies reported no significant effects of the intervention on self-efficacy [18, 30]. Draffin et al. study concluded non-significant results were due to pregnant women frequently seeing healthcare providers and being well-supported by family and friends; thus affecting their self-efficacy positively, and self-managing their condition [18]. Sen and Sirin study had an intervention duration of two hour session over four consecutive days [30]. A limitation of this study may be the short intervention duration with minimal time to produce an effect on the self-efficacy levels of women. This limitation is supported by other studies that showed positive effects on self-efficacy occurred when the duration of intervention ranged between four and seven weeks [16, 19, 21].

#### Effects of interventions on self-care behaviour

As shown in Table 4, five studies examined self-care behaviour as an outcome using various measurements. Four studies reported statistically significant differences on self-care behaviour for the intervention group compared to the control group [16, 25, 30, 31]. One study reported a positive impact of the intervention but did not reach a statistical significance [28]. The consistent positive findings in these studies may be due to the content of educational programs, which focused on self-care information and supported women to improve their self-care behaviour, such as setting goals and glucose monitoring.

#### Effects of interventions on depression and anxiety

Depression, anxiety, and stress were measured in four studies via different measurement tools [18, 24, 26, 28]. Draffin et al. study reported no significant differences between groups in levels of anxiety [18]. The authors concluded that pregnant women were frequently assessed by a diabetes care team which might have positively reduced anxiety levels [18]. Kim et al. study found no significant differences between intervention and control groups as both groups demonstrated increased levels of depression compared to baseline data, with no significant differences between the two groups [28]. However, content of the intervention did not include information about depression, and symptoms of depression; thus, there was no impact on the participants’ level of depression. This study also found that anxiety levels decreased in the intervention group and increased in the control group, but the difference was insignificant. Zaheri et al. study reported that depression and anxiety scores were significantly lower in the intervention group compared with the control group [24]. The stress management intervention provided different measures, such as coping skills, coping with stress advice, progressive relaxation techniques, problem-solving skills, and anger management [24]. Zeinabeh et al. study also reported mindfulness training sessions significantly reduced stress level in the intervention group [26].

#### Effects of interventions on birthing outcomes

Two studies considered preterm birth as an outcome; but found no significant differences between the intervention and control groups [20, 27]. These studies concluded that future research require a larger sample size. Whereas three studies included birth type as the outcome. One study found significantly fewer caesarean births in the intervention group compared with the control group [20]. Two studies reported no statistically significant difference between groups [19, 27]. One study concluded that a larger sample size is needed to achieve statistically significant differences between groups [27]. The other study attributed the lack of statistically significant differences to the high rate of previous caesarean births in participants in both groups [19]. Infant birth weights were measured in seven studies. Two studies reported statistically significant differences between groups with higher birth weights in the control group [20, 23], and the remaining found no significantly different results between intervention and control groups [17, 19, 27, 29]. Three studies included Neonatal Intensive Care Unit (NICU) admission as an outcome. One study examined the effects of a self-care package including; DVDs, self-care guidebook, software, a logbook and face-to-face educational sessions, found that NICU admission rates were higher in the control group than in the intervention group [19]. The other two studies reported no statistically significant difference between groups [27, 29].

#### Theoretical frameworks

The review found only four studies that used a theoretical framework to support their interventions [21, 29–31]. Social Cognitive Theory (SCT) and Health Promotion Model (HPM) were used both together in two studies by the same authors who reported similar interventions but different outcomes [29, 30]. SCT focuses on the mutual interaction between a person’s behaviour and environment. It aims to help individuals change their behaviour through self-control and reinforcement to commence goal-oriented behaviours that can be maintained for longer periods [33]. The HPM model focuses on three important concepts: individual characteristics and experiences, behaviour-specific cognition and affect, and behavioural outcomes. Mohebbi et al. study was based on the Health Belief Model (HBM) which focuses on psychosocial health behaviour changes [21]. It explains the relationship between individuals’ health beliefs and their health behaviours. It comprises six elements: perceived susceptibility to disease, perceived disease severity, perceived benefits of action, perceived barriers preventing action, motivation behind an action, and self-efficacy [34]. Orem’s nursing model theory was used in a study by Viswanath and Jose study [31]. The model comprises of three interrelated parts: the theory of self-care, self-care deficit, and the nursing system.

## Discussion

This integrative literature review synthesised research evidence on the effectiveness of psychoeducational interventions on GDM knowledge, self-efficacy, self-care behaviour, depression, anxiety, and birth outcomes among pregnant women with GDM. Mixed results were found, which may be attributed to variations in the intervention content, duration, and frequency. Furthermore, different tools were used to measure similar outcomes, making it difficult to compare these studies.

Face-to-face educational interventions showed positive outcomes regarding women’s knowledge of GDM. This educational intervention provided information about GDM and management, and used different strategies, such as group discussions, sharing experiences, and booklets [29]. Face-to-face interventions facilitated knowledge acquisition by allowing women to receive advice tailored to their specific needs and levels. Specifically, in small groups of five to ten women. This review found multi-component educational interventions may meet various learning styles and needs of pregnant women with GDM. Thus, a range of different teaching strategies should be used when designing educational interventions for women with GDM.

Studies that used web- and DVD-based methods to deliver knowledge found no significant differences between educational intervention and control groups compared to studies where participants received face-to-face health education. Both studies provided similar knowledge but used different delivery methods [18, 22]. As neither of the studies measured baseline knowledge, there was no comparison between the change in knowledge before and after the intervention. Future studies should measure women’s knowledge at baseline and after the intervention. One of the limitations of face-to-face delivery is that not all pregnant women have the opportunity or time to attend sessions [35].

The current COVID-19 pandemic is a challenge for face-to-face educational programs. Online web, video, and DVD-based methods have advantages, such as accessibility, convenience, and satisfaction. As such there is a need to develop alternative ways to support women with GDM, such as using a blended intervention approach integrating face-to-face whereas possible with internet-based interventions. Blended interventions also provide a time-efficient and cost-effective approach as online complements to face-to-face interventions can be an effective way to deal with problems proactively [36]. This approach has positive effects on mental health care [37], and can be adopted for pregnant women with GDM.

Regarding the self-efficacy of pregnant women, studies that showed positive outcomes used multi-component intervention strategies with various educational methods, such as formulating individual goals, group discussions, sharing experiences, videos, and phone calls as booster sessions to support and motivate the participants [16, 19, 21]. Multi-component strategies effectively enhance women’s self-efficacy levels more than single-component interventions. We found two RCTs [18, 19], and three were quasi-experimental studies [16, 21, 30] that measured women’s self-efficacy, however, more rigorous RCTs are needed to improve the quality of the evidence.

This review found consistent positive results regarding the effects of educational interventions on women’s self-care behaviours. These findings were possibly due to the strategies used in the educational interventions, such as individual goal setting, recording blood glucose readings, practising using an insulin pen, sharing experience, and filling dietary logs [16, 25, 28, 30, 31]. Future studies should consider similar strategies to encourage, motivate, and support women during pregnancy. From findings, it is vital to provide regular interventions to encourage changes in self-care behaviours in women with GDM. Therefore, we recommend that minimum of four sessions, each lasting 30 min or more, can effectively change self-care behaviours.

Regarding depression, anxiety and stress levels, two studies reported positive impacts used cognitive behavioural stress management training and mindfulness training to reduce depression and anxiety levels among women with GDM [24, 26]. Those interventions included stress-coping styles, anger management, problem-solving skills, information about the relationship between stress and GDM, and relaxation techniques such as meditation, mindfulness and breathing exercises. However, studies that reported no statistically significant differences in depression and anxiety levels did not provide women with information about the mental state and relaxation techniques used to cope with stress during pregnancy [18, 28]. Women with GDM are categorised as high-risk patients for adverse obstetric and perinatal outcomes; therefore, supporting them women physically and psychologically is crucial. Therefore, future studies should include strategies to support the mental and emotional state of women with GDM.

Theoretical framework was not an identified outcome measure; however, the present review found four studies that adopted a theoretical framework to develop the interventions, though the frameworks used, varied [21, 29–31]. Theories are important to support the development of health interventions to guide and improve future health practices, and to generalise and replicate interventions across cultures [38]. However, these theoretical frameworks have certain limitations. For example, HBM theory does not provide strategies for changing behaviour [39]. Furthermore, the HPM theory focuses more on the promotion of health and the prevention of disease [40].

Self-management of diabetes is critical for achieving positive health outcomes. A sense of self-efficacy or confidence in one’s ability to control blood glucose levels is essential for successful self-management. Self-efficacy beliefs determine how people think, feel, act, and encourage themselves; thus, they are the essential precondition for changing behaviour [41]. Studies have shown that self-efficacy is crucial in disease self-management [42, 43]. Furthermore, self-efficacy is directly proportional to self-management. Thus, future studies should consider theories of change as a framework to support the development of self-care, such as Bandura’s Self-Efficacy Theory, to empower pregnant women to manage GDM.

Most studies reviewed used quantitative methods. Only one study used a mixed-methods approach, interviewing study participants to evaluate the impact of the educational intervention [31]. This study however, did not analyse qualitative data. Furthermore, none of the included studies mentioned whether the interventions were developed based on women’s needs or involvement. Mixed-methods research provides a comprehensive account of the issues being studied [44]. With a clear lack of research using a mixed-method approach, future studies with mixed-methods designs are necessary to gain an in-depth understanding of women’s needs and concerns and to produce persuasive evidence for clinical decisions.

In this review, a duration of four weeks was the most common duration for the interventions [16, 19, 21, 25, 26, 31]. This duration seems acceptable for pregnant women with GDM. However, not all studies reported the duration of each session. Thus, future studies should report the number and duration of sessions to guide healthcare organisations and professionals when developing new interventions. Furthermore, this review found that informational support and encouragement interventions for self-management are frequently used in GDM management. Only one study provided informational support, self-management encouragement, emotional support, and relaxation techniques. A lack of GDM knowledge and motivation can make self-management difficult. In addition, controlling GDM is stressful for pregnant women. Thus, future research should provide informational support and motivational interventions for women with GDM and integrate relaxation and emotional support elements.

### Strengths and limitations

The strength of this review is the use of the PRISMA guidelines and a standard methodological quality checklist to evaluate and present evidence. Furthermore, it followed Whittemore and Knafl’s methodology to identify and ensure reliable results (Whittemore & Knafl, 2005). However, this review has one limitation. Only studies published in English language were included. Although the inclusion criteria were designed to include studies in Arabic, there were no eligible studies found either in Arabic or from Saudi Arabia; this is an identified gap in the literature and needs to be addressed.

## Conclusion

This integrative review reports some educational interventions can improve pregnant women’s self-efficacy, knowledge, and self-care behaviour and reduce depressive symptoms among women with GDM. Educational interventions providing informational support and motivation should continue to support women with GDM. However, the identified gap is that relaxation techniques and emotional support interventions to reduce stress, anxiety and depression are scarcely used in pregnant women with GDM. Psychosocial elements should be integrated into the educational content using a holistic care approach. Therefore, future studies should consider holistic care approach and multi-component psychoeducational interventions using a blended delivery approach.

## Data Availability

The data that support the findings of this study are available from the corresponding author upon reasonable request.
